# Administration of Noggin Suppresses Fibrinogen Leakage into the Brain in the Acute Phase After Traumatic Brain Injury in Mice

**DOI:** 10.3390/ijms26073002

**Published:** 2025-03-25

**Authors:** Miho Yasunaga, Fuyuko Takata, Takuro Iwao, Junko Mizoguchi, Nanako Tajima, Shinya Dohgu

**Affiliations:** Department of Pharmaceutical Care and Health Sciences, Faculty of Pharmaceutical Sciences, Fukuoka University, Fukuoka 814-0180, Japan; pd211010@cis.fukuoka-u.ac.jp (M.Y.); t.iwao.ot@adm.fukuoka-u.ac.jp (T.I.); pd231004@cis.fukuoka-u.ac.jp (J.M.); pp190096@cis.fukuoka-u.ac.jp (N.T.); dohgu@fukuoka-u.ac.jp (S.D.)

**Keywords:** traumatic brain injury, noggin, fibrinogen leakage, bone morphogenetic protein 4, neurovascular unit, neuronal damage

## Abstract

Traumatic brain injury (TBI) causes neurovascular unit (NVU) dysfunction, including hyperpermeability of the blood–brain barrier to fibrinogen, glial activation, and neuronal damage, possibly leading to secondary brain damage. However, no known substance can inhibit its pathogenesis. In this study, we investigated noggin, a bone morphogenetic protein (BMP) 4 inhibitor, as a TBI pathogenesis-inhibiting substance. We induced acute TBI in C57BL/6J mice through a controlled cortical impact (CCI) and evaluated the effects of noggin on fibrinogen leakage into the brain and NVU-constituting cells, including pericytes, microglia, astrocytes, and neurons. CCI mice showed increased BMP4 levels and extravascular fibrinogen in the hippocampus. Noggin treatment significantly suppressed fibrinogen leakage four days post-CCI in a dose-dependent manner. Immunofluorescence staining revealed that noggin administration did not inhibit the activation of NVU cells such as pericytes, microglia, and astrocytes, which were characterized by increased PDGFRβ, Iba1, and GFAP expression levels, respectively. On postoperative day 4, CCI mice showed neuronal cell and myelinated neuronal fiber loss, which were not significantly affected by noggin administration. In conclusion, noggin administration suppresses fibrinogen leakage into the brain in the acute phase after TBI. However, the suppression of fibrinogen leakage through noggin administration did not alleviate neuronal damage and activation of NVU cells during the acute phase of TBI.

## 1. Introduction

Traumatic brain injury (TBI), caused by traffic accidents, falls, and some contact sports [1,2,3,4], is a risk factor for secondary brain damage, including neurodegenerative diseases [5,6,7]. TBI induces the activation of cell types constituting the neurovascular unit (NVU), such as astrocytes, microglia, and pericytes, as well as neuronal damage [8,9,10]. The characteristic reactions of the NVU cell types probably drive secondary brain damage [11,12]. TBI impairs the function of the blood–brain barrier (BBB), a crucial component of the NVU, leading to extravascular leakage of plasma-derived fibrinogen into the brain parenchyma. It also contributes to neuronal inflammation and damage in neurodegenerative diseases [13]. However, TBI-induced astrocyte activation and neuronal inflammation have been inhibited by genetically knocking down fibrinogen in mice [8], suggesting that reducing fibrinogen leakage prevents NVU cell activation and neuronal damage. Nevertheless, no therapeutic agent effectively inhibits TBI-induced fibrinogen leakage.

Noggin is an endogenous protein that inhibits bone morphogenetic proteins (BMPs), which belong to the TGF-β superfamily. TBI increases BMP4 levels and reduces noggin levels in the brain [14,15], suggesting that the BMP4 pathway is upregulated in the brain following TBI. BMP4 reduces endothelial barrier function [16], inhibits neurogenesis [17], and increases astrocyte density in the brain [18]. This suggests that increased BMP4 levels contribute to fibrinogen leakage, NVU cell activation, and neuronal damage in the brain following TBI. Incidentally, noggin overexpression in transgenic mice has been shown to reduce ischemic brain injury after permanent middle cerebral artery occlusion [19]. Noggin administration rescues age-related neuronal stem cell loss and age-related decline in neurogenesis [20]. The neuroprotective role of noggin has been reported in animal models of spinal cord injury [21]. These findings suggest that noggin plays a neuroprotective role under pathological conditions. However, there is no evidence indicating that noggin administration suppresses TBI-induced pathological changes.

This study aimed to investigate whether noggin administration reduces fibrinogen leakage and, if so, whether this reduction alleviates NVU cell activation and neuronal damage in the mouse brain during the acute phase after TBI, as assessed through histological analyses.

## 2. Results

### 2.1. Effect of Noggin Administration on Fibrinogen Leakage into the Ipsilateral Hippocampus of CCI Mice Showing Increased BMP4 Levels

**Figure 1:**
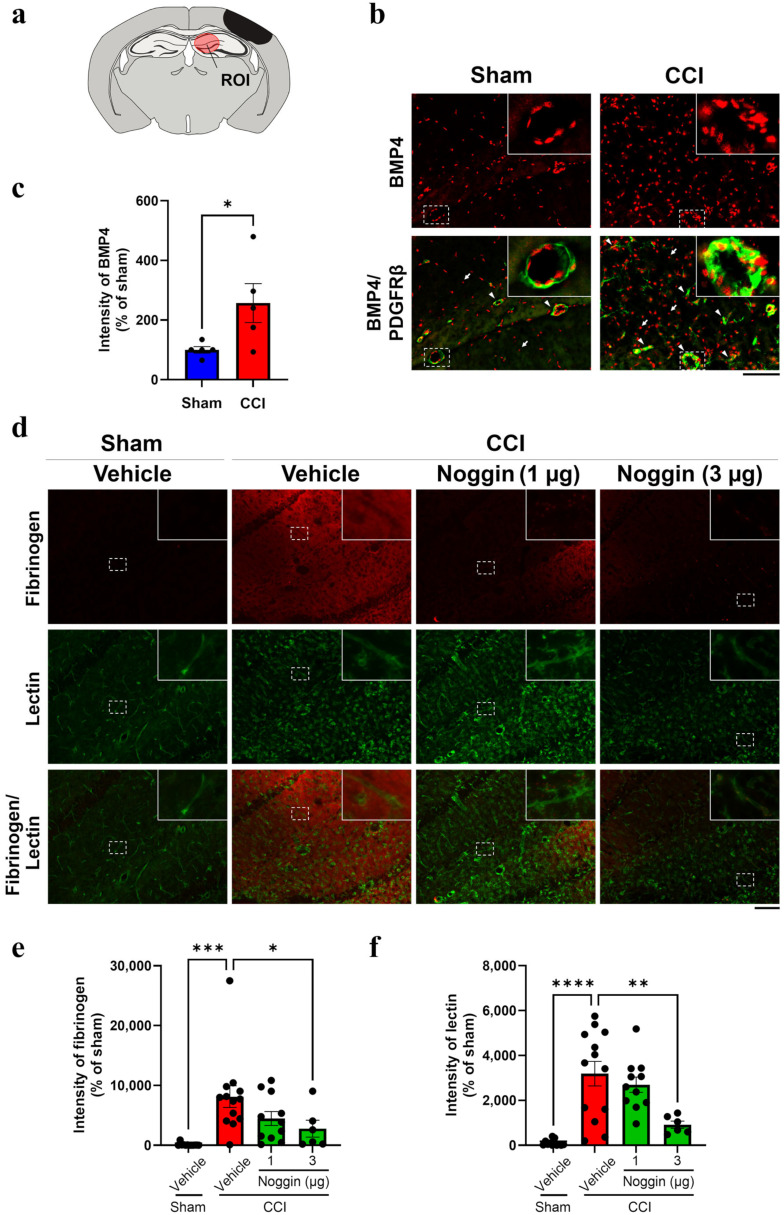
BMP4 immunoreactivity in the ipsilateral hippocampus of controlled cortical impact (CCI) mice and effect of noggin on extravascular fibrinogen leakage on postoperative day 4. (**a**) The brain diagram shows the lesion and region of interest (ROI), including the hippocampus CA1 and dentate gyrus. (**b**) The top panels show bone morphogenetic protein (BMP) 4 immunoreactivity (red) in sham and CCI mice on postoperative day 4. The bottom panels show merged BMP4 (red) and PDGFRβ (green) immunoreactivity images. The arrowheads and arrows in the bottom panels indicate BMP4 immunoreactivity in the brain vessels and parenchyma, respectively. Scale bar: 100 μm. (**c**) Quantification of BMP4 immunoreactivity. Sham: *n* = 5, CCI: *n* = 5. The blue and red bars represent the sham and CCI group, respectively. Each point represents an individual mouse. Data are expressed as the mean ± SEM. (**d**) CCI mice were treated with noggin at 1 or 3 μg/mouse for 4 days from the operative day to postoperative day 3. The top panels show fibrinogen immunoreactivity (red). The middle panels show brain vessels revealed through lectin staining (green). Each bottom panel shows the merged image of the top and middle panels. Scale bar: 100 μm. (**e**) Quantification of fibrinogen immunoreactivity. Vehicle + sham: *n* = 12, vehicle + CCI: *n* = 13, 1 μg noggin + CCI: *n* = 11, 3 μg noggin + CCI: *n* = 6. (**f**) Quantification of lectin intensity. Vehicle + sham: *n* = 12, vehicle + CCI: *n* = 13, 1 μg noggin + CCI: *n* = 11, 3 μg noggin + CCI: *n* = 6. The blue, red and green bars represent the vehicle + sham, vehicle + CCI and noggin + CCI groups, respectively. Each point represents an individual mouse. Data are expressed as the mean ± SEM. * *p* < 0.05, ** *p* < 0.01, *** *p* < 0.001, **** *p* < 0.0001.

Four days post-surgery, BMP4 immunoreactivity was markedly increased in the ipsilateral hippocampus of controlled cortical impact (CCI) mice (top right panel of Figure 1b), whereas sham mice exhibited low-intensity dispersed BMP4 signals in the same region in (top left panel of Figure 1b). Some BMP4 immunoreactive signals were merged to the platelet-derived growth factor receptor (PDGFR) β-immunoreactive pericytes in the perivascular area (bottom right panel of Figure 1b). Quantitative analysis showed that BMP4 intensity in the ipsilateral hippocampus of CCI mice was 2.57-fold higher than that in sham mice (Figure 1c, *p* < 0.05). To detect extravascular fibrinogen leakage, mouse brain sections were co-stained for fibrinogen and lectin (a vascular marker). Intense fibrinogen immunoreactivity was observed in the ipsilateral hippocampus of CCI mice with no colocalization with lectin, indicating fibrinogen leakage from the blood vessels (Figure 1d). The intensity of fibrinogen immunoreactivity in CCI mice was significantly higher (81.2-fold) than that in sham mice (Figure 1e). Noggin (1 or 3 µg/mouse) was administered to mice from the day of surgery until postoperative day 3. The treatment reduced the fibrinogen leakage in a dose-dependent manner by 44.8 (*p* = 0.12) and 65.8% (*p* < 0.05) in CCI mice receiving 1 and 3 µg, respectively, compared to vehicle-treated CCI mice (Figure 1d,e). Lectin intensity, which was significantly higher in CCI mice (31.9-fold vs. sham, Figure 1f), decreased by 71.2% following the administration of 3 µg noggin (Figure 1d,f).

### 2.2. Effects of Noggin on the Activation of NVU-Constituting Cell Types in CCI Mice

**Figure 2:**
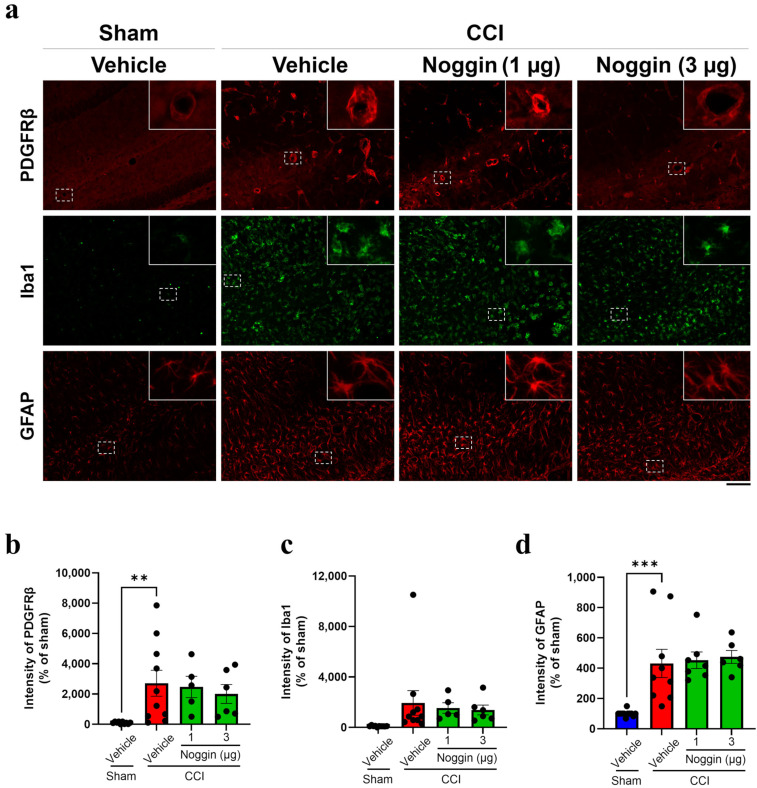
Effect of noggin on the PDGFRβ, Iba1, and GFAP expression levels in the ipsilateral hippocampus of controlled cortical impact (CCI) mice on postoperative day 4. (**a**) The top panels show PDGFRβ immunoreactivity (red). The middle panels show Iba1 immunoreactivity (green). The bottom panels show GFAP immunoreactivity (red). Scale bar: 100 μm. (**b**) Quantification of PDGFRβ immunoreactivity, vehicle + sham: *n* = 10, vehicle + CCI: *n* = 10, 1 μg noggin + CCI: *n* = 5, 3 μg noggin + CCI: *n* = 6. (**c**) Quantification of Iba1 immunoreactivity, vehicle + sham: *n* = 10, vehicle + CCI: *n* = 10, 1 μg noggin + CCI: *n* = 5, 3 μg noggin + CCI: *n* = 6. (**d**) Quantification of GFAP immunoreactivity. Vehicle + sham: *n* = 11, vehicle + CCI: *n* = 9, 1 μg noggin + CCI: *n* = 7, 3 μg noggin + CCI: *n* = 6. CCI mice were treated with noggin at 1 or 3 μg/mouse for 4 days from the operative day to postoperative day 3. The blue, red and green bars represent the vehicle + sham, vehicle + CCI and noggin + CCI groups, respectively. The point represents an individual mouse. Data are expressed as the mean ± SEM. ** *p* < 0.01, *** *p* < 0.001.

The activation of pericytes, microglia, and astrocytes was assessed by determining immunoreactivity for PDGFRβ, ionized calcium-binding adaptor protein 1 (Iba1), and glial fibrillary acidic protein (GFAP). CCI mice showed an increase in PDGFRβ, Iba1, and GFAP-positive areas compared to sham mice (Figure 2a). The intensity of PDGFRβ, Iba1, and GFAP immunoreactivity was increased by 27.1—(*p* < 0.01), 19.4—(*p* = 0.09), and 4.32—fold (*p* < 0.001) in vehicle-treated CCI mice (Figure 2b–d). However, PDGFRβ, Iba1, and GFAP immunoreactivity did not change significantly in CCI mice treated with noggin at 1 or 3 µg compared with that in vehicle-treated CCI mice (Figure 2a–d), indicating that CCI-activated pericytes, microglia, and astrocytes were hardly affected by noggin administration.

### 2.3. Effects of Noggin on Neuronal Damage in CCI Mice

**Figure 3:**
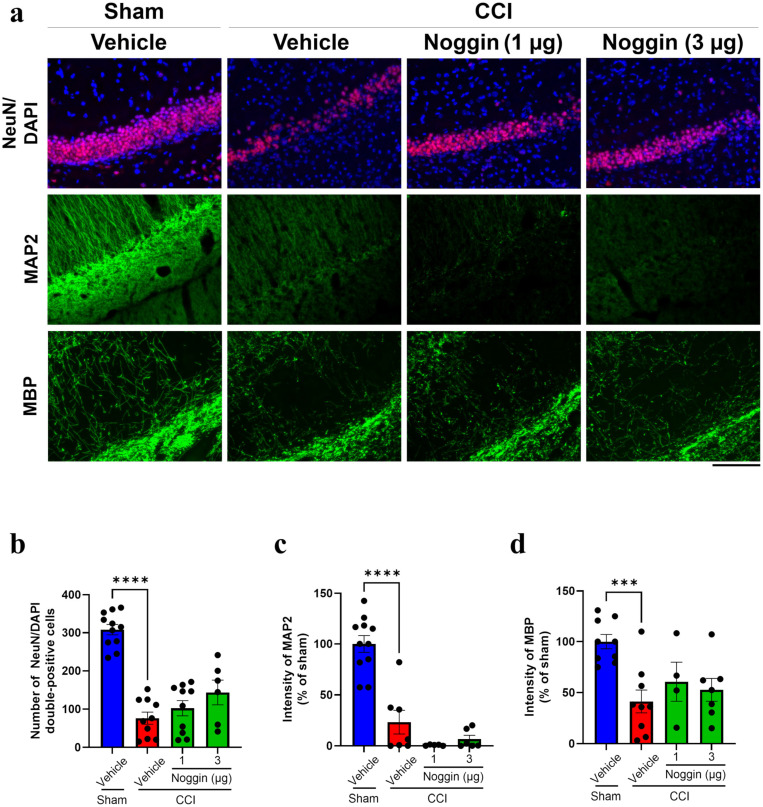
Effect of noggin on NeuN, MAP2, and MBP expression levels in the ipsilateral hippocampus of controlled cortical impact (CCI) mice on postoperative day 4. (**a**) The top panels show images of NeuN (red) and DAPI (blue) double-labeled staining. The middle panels show MAP2 immunoreactivity (green). The bottom panels show MBP immunoreactivity (green). Scale bar: 100 μm. (**b**) The number of NeuN/DAPI double-positive cells. Vehicle + sham: *n* = 11, vehicle + CCI: *n* = 10, 1 μg noggin + CCI: *n* = 10, 3 μg noggin + CCI: *n* = 6. (**c**) Quantification of MAP2 immunoreactivity. Vehicle + sham: *n* = 11, vehicle + CCI: *n* = 7, 1 μg noggin + CCI: *n* = 5, 3 μg noggin + CCI: *n* = 6. (**d**) Quantification of MBP immunoreactivity. Vehicle + sham: *n* = 9, vehicle + CCI: *n* = 9, 1 μg noggin + CCI: *n* = 4, 3 μg noggin + CCI: *n* = 7. CCI mice were treated with 1 or 3 μg/mouse for 4 days from the operative day to postoperative day 3. The blue, red and green bars represent the vehicle + sham, vehicle + CCI and noggin + CCI groups, respectively. Each data point represents an individual mouse. Data are expressed as the mean ± SEM. *** *p* < 0.001, **** *p* < 0.0001.

To evaluate the effect of noggin on neuronal cell loss in the ipsilateral hippocampus of CCI mice, brain sections were stained with anti-neuronal nuclei (NeuN) antibody and DAPI (top panel, Figure 3a). In vehicle-treated CCI mice, NeuN-DAPI double-positive cells were markedly reduced, and noggin administration (3 µg/mouse) did not restore this loss (Figure 3b). To evaluate the effect of noggin on neuronal fiber loss in the ipsilateral hippocampus of CCI mice, brain sections were stained for microtubule-associated protein 2 (MAP 2) and myelin basic protein (MBP) (Figure 3a, middle and bottom panels). In vehicle-treated mice, MAP2 and MBP immunoreactivity significantly decreased to 23.3 and 41.4% of sham levels, respectively, and noggin administration failed to restore these reductions (Figure 3c,d).

## 3. Discussion

This study demonstrated that noggin administration suppressed extravascular fibrinogen leakage in the ipsilateral hippocampus of CCI mice on postoperative day 4. However, despite reducing fibrinogen leakage, noggin administration did not alleviate neuronal damage, such as the loss of neuronal cells, filaments, or myelin, and did not inhibit the activation of NVU cells, such as pericytes, microglia, or astrocytes, in CCI mice on postoperative day 4.

Among several routes of administration, such as intravenous, intraperitoneal, subcutaneous, and intranasal injection, intranasal administration has been reported as the most effective for delivering protein to the brain [22]. Additionally, topical application of therapeutic materials to the cortical surface facilitates damage repair in the brain [23,24]. Therefore, in this study, noggin solution was administered to CCI mice by intranasal route and by topical application to the surface of the injured cortex.

Administration of noggin at 1 µg/day has been reported to reduce neurological deficit in mice with ischemic brain injury [25]. Based on this study, we decided to set the minimum dose of noggin at 1 µg to evaluate the effect of noggin on fibrinogen leakage in CCI mice. The noggin administration at 3 µg suppressed fibrinogen leakage but did not alleviate the NVU activation and neuronal damage in the acute phase. These findings indicated that the concentration of noggin delivered around brain vessels of the brain parenchyma is enough to suppress the CCI-induced fibrinogen leakage and that the suppression of fibrinogen leakage through noggin administration did not lead to inhibition of NVU activation and neuronal damage in the acute phase. In other words, NVU activation and neuronal damage in the acute phase (at 4 days after CCI) may not be triggered by CCI-induced fibrinogen leakage. However, noggin has been reported to have abilities to promote neurogenesis and reduce astrogliogenesis in the brain [26,27]. Therefore, to evaluate whether the noggin level around neurons in the brain parenchyma is sufficient to exert these effects, further studies should measure noggin concentration in the brain parenchyma.

Administering noggin to CCI mice until postoperative day 3 significantly reduced fibrinogen leakage on postoperative day 4. Since TBI-induced BBB impairment causes extravascular fibrinogen leakage, noggin may prevent BBB dysfunction during the acute phase of TBI. Vehicle-treated CCI mice exhibited a significant increase in the lectin signal, indicating increased capillary density in the brain. This aligns with reports that post-TBI angiogenesis increases vascular density [28], which may contribute to BBB dysfunction [29,30]. Noggin treatment significantly suppressed the increased lectin signal in CCI mice, suggesting a possibility that the suppression of TBI-induced angiogenesis through noggin administration might prevent BBB dysfunction. Noggin is a known antagonist of BMPs, including BMP4 [31]. In the present study, BMP4 levels were elevated in the brain vasculature of CCI mice. Endothelial cell-derived BMP4 has been linked to barrier dysfunction characterized by decreased cadherin expression in endothelial cells [16]. Our findings suggested that noggin may alleviate BBB dysfunction in CCI mice caused by increased BMP4 induced by the cell types constituting brain vessels, including brain endothelial cells and pericytes, resulting in fibrinogen leakage suppression. A previous study showed increased BMP7 expression in the brains of TBI mice [14]. Noggin inhibits BMP7 and BMP4 [31]. Thus, noggin may antagonize the expression of BMP4 and BMP7 during TBI. Further investigations are required to elucidate the precise mechanisms by which noggin administration suppresses TBI-induced extravascular fibrinogen leakage in the acute phase.

Fibrinogen leakage into the brain has been reported to activate glial cells and induce neuronal damage [8,13,32,33,34,35,36]. NVU cells, including pericytes, microglia, and astrocytes, are activated following TBI, contributing to neural dysfunction [8,9,10]. Managing NVU activation and neuronal damage is crucial for mitigating TBI-induced neurological dysfunction. Our findings indicated that despite reducing fibrinogen leakage, noggin treatment did not suppress NVU activation or neuronal damage at four days post-CCI. This suggests that NVU activation and neuronal damage in the acute phase of TBI may be driven by factors other than elevated fibrinogen in the brain. A previous study has suggested that astrocyte activation and neuronal damage in the acute phase could be induced by the deformation of cell membranes due to physical strain resulting from head impact [37,38]. This finding suggests that noggin does not alleviate the activation of NVU cells and neuronal damages, because mechanical stress, rather than fibrinogen accumulation, is the primary driver of acute phase damages in TBI.

Our study has limitations. We did not evaluate the effect of noggin on NVU activation, neuronal damage, or cognitive impairment in the chronic phase of TBI. Several clinical studies showed that TBI can cause neurological disorders such as Alzheimer’s and Parkinson’s diseases several years after the initial brain injury [7]. Persistent fibrinogen leakage has been observed in the brains of patients with TBI [39]. In addition, fibrinogen leakage into the brain triggers microglial activation, axonal loss, and cognitive dysfunction, which are the pathological hallmarks of Alzheimer’s disease [32,33]. These findings suggest that inhibiting fibrinogen leakage in the acute phase of TBI may prevent the development of neurological dysfunction in the chronic phase. Therefore, future studies should investigate the therapeutic effects of fibrinogen leakage inhibition by noggin on the activation of NVU cells, neuronal damage, and impaired cognitive function in the chronic phase of TBI.

## 4. Materials and Methods

### 4.1. Animals

Male C57BL/6J mice (6–8 weeks old; Jackson Laboratory Japan, Yokohama, Japan) weighing 18–23 g were used. Mice were housed at a constant temperature (24 ± 1 °C) and humidity (50 ± 5%) under a 12-h light/dark cycle and were given food and water ad libitum.

### 4.2. Preparation of a TBI Mouse Model Through CCI

The CCI procedures have been previously described [9]. Briefly, mice were anesthetized with isoflurane, the skull was exposed through a midline scalp incision, and a 4-mm craniotomy was made at −2.5 mm from bregma and 2.75 mm lateral to the midline on the left hemisphere. CCI models of TBI were constructed using a device comprising a computer-controlled pneumatic impactor (AMS 201, AmScien Instruments, Richmond, VA, USA) with a 3-mm flat tip. The punch depth was set at 0.5 mm using a screw-mounted adjustment. CCI was performed at a speed of 3.0 m/s and a 120 ms duration. After the CCI injury, the wound was sutured, and the mice were placed on a heating pad to maintain a normal body temperature until they were fully awake. Sham-operated control mice underwent all surgical procedures except CCI injury.

### 4.3. Administration of Noggin

Recombinant noggin (#6057-NG; R&D Systems, Minneapolis, MN, USA) was dissolved in sterilized, phosphate-buffered saline (PBS), supplemented with 0.1% bovine serum albumin to prepare the noggin solution. Thereafter, noggin solutions (1 or 3 µg/40 µL of solvent) were administered into the nasal cavity of mice under inhalation anesthesia with isoflurane. This was completed using a micropipette, alternating nostrils approximately 25 times. Intranasal administration was performed an hour before CCI and every 24 h until 3 days after CCI. In addition, noggin solutions (1 or 3 µg/4 µL of solvent) were instilled into the cortical injury site immediately after CCI. In the vehicle group, mice were treated with the same solvent volume using the same methods as in the noggin group.

### 4.4. Histological Analyses

#### 4.4.1. Sample Preparation

Four days after injury, mice were deeply anesthetized with isoflurane and transcardially perfused with cold PBS, followed by cold 4% paraformaldehyde (PFA). The brains were carefully removed from the skull, fixed in 4% PFA overnight at 4 °C, and subsequently placed in 20% sucrose dissolved in PBS until the brain tissue was equilibrated. Thereafter, brains were covered with an optimal cutting temperature compound (Sakura Finetek Japan Co., Ltd. Tokyo, Japan) and frozen using dry ice. The embedded brains were sectioned serially using a cryostat (−20 °C) at a thickness of 20 μm (CM1850, Leica, Wetzlar, Germany).

#### 4.4.2. Immunohistochemical Assay

Immunohistochemical staining was performed as previously described [9]. Briefly, coronal brain cryosections were rinsed in 0.2% Triton-X100 (Sigma-Aldrich, St. Louis, MO, USA) in Tris-buffered saline (T-TBS) and blocked with Blocking One Histo (#06349-64; Nacalai Tesque, Kyoto, Japan). Afterward, the sections were incubated with primary antibodies: rabbit anti-fibrinogen (1:1000, #A0080; Dako, Santa Clara, CA, USA), rabbit anti-BMP4 (1:400 #ab39973 Abcam, Cambridge, UK), goat anti-PDGFRβ (1:100, #AF1042; R&D Systems), rabbit anti-GFAP (1:500, #AB5804; Merck Millipore, Burlington, MA, USA), rabbit anti-Iba1 (1:100, #019-19741; FUJIFILM Wako Pure Chemical Corporation, Osaka, Japan), rabbit anti-NeuN (1:200, #24307S; Cell Signaling Technology, Danvers, MA, USA), rabbit anti-MBP (1:100, #ab40390; Abcam), and rabbit anti-MAP2 (1:2500, #17490-1-AP; Proteintech Group, Inc., Rosemont, IL, USA) in 5% Blocking One Histo in T-TBS overnight at 4 °C. Subsequently, the slices were washed with T-TBS and incubated with respective secondary antibodies for 2 h at room temperature. The secondary antibodies used were donkey anti-goat Cy3 (1:500, #705-165-147; Jackson ImmunoResearch, West Grove, PA, USA), donkey anti-rabbit Alexa Fluor 488 (1:500, #A-21206; Thermo Fisher Scientific, Waltham, MA, USA), donkey anti-rabbit CY3 (1:200, #711-165-152; Jackson ImmunoResearch), and lectin (1:200, #DL-1174; Vector Laboratories, Newark, CA, US). After washing in T-TBS, the slices were mounted using Vectashield containing DAPI (#H-1200; Vector Laboratories). The brain sections subjected to immunohistochemical staining were imaged using a BZ-X710 microscope (Keyence, Osaka, Japan). The region of interest (ROI), including the hippocampus CA1 and dentate gyrus areas, were observed (approximately −1.8 mm to −2.0 mm from bregma) (Figure 1a). The fluorescence intensity of each target protein (BMP4, fibrinogen, PDGFRβ, Iba1, GFAP, MAP2, and MBP) was quantified using the BZ-X Analyzer 1.4.1.1 software (Keyence). The data were expressed as the average fluorescence intensity per square micrometer of ROI in arbitrary units. The number of NeuN-DAPI double-positive cells was counted in the ROI with the same-sized field of view, and the data were expressed as the average number of cells.

### 4.5. Statistical Analysis

Statistical analyses were performed using GraphPad Prism 10.4.1 software (GraphPad Software, San Diego, CA, USA). Differences among more than two groups were assessed using ANOVA, followed by Dunnett’s test for multiple comparisons. To compare between two groups, significant differences were assessed using Student’s *t*-test. Results were expressed as mean ± standard error of the mean (SEM), and differences were considered statistically significant if the *p*-values were < 0.05.

## 5. Conclusions

In this study, we demonstrated that noggin administration suppressed fibrinogen leakage in the ipsilateral hippocampus during the acute phase of TBI. However, fibrinogen leakage inhibition through noggin administration did not alleviate neuronal damage and NVU cell activations in the ipsilateral hippocampus of TBI mice during the acute phase. This study provides novel insights into the effects of noggin on fibrinogen infiltration in the brain of TBI mice.

## Data Availability

The original contributions presented in this study are included in the article material. Further inquiries can be directed to the corresponding author.

## Abbreviations

The following abbreviations are used in this manuscript:

BBB	blood–brain barrier
BMP	bone morphogenetic protein
CCI	controlled cortical impact
GFAP	glial fibrillary acidic protein
Iba1	ionized calcium-binding adaptor protein 1
MAP2	microtubule-associated protein 2
MBP	myelin basic protein
NeuN	neuronal nuclei
NVU	neurovascular unit
PBS	phosphate-buffered saline
PDGFRβ	platelet-derived growth factor receptor β
TBI	traumatic brain injury
TGF	transforming growth factor
